# Interpersonal violence-related physical injury in low- and middle-income countries and its association with markers of socioeconomic status: a systematic review

**DOI:** 10.1186/s12889-025-21321-6

**Published:** 2025-03-19

**Authors:** Kevin J. Blair, Haley Tupper, Jordan M. Rook, Michael de Virgilio, Thiago S. Torres, Akshayaa K. Chittibabu, M. Wynn Tranfield, Bethany Myers, Alan Hubbard, Alain Chichom-Mefire, Mary-Margaret Ajiko, Catherine Juillard, Rochelle Dicker, Fanny Nadia Dissak-Delon

**Affiliations:** 1 Program for the Advancement of Surgical Equity, Department of Surgery, University of California Los Angeles (UCLA), Los Angeles, CA USA; 2 Department of Surgery, University of California Irvine (UCI), Irvine, CA USA; 3 Instituto Nacional de Infectologia Evandro Chagas, Fundação Oswaldo Cruz (INI-Fiocruz), Rio de Janeiro, RJ Brasil; 4 David Geffen School of Medicine, UCLA, Los Angeles, CA USA; 5 Louise M. Darling Biomedical Library, UCLA, Los Angeles, CA USA; 6 Division of Biostatistics, School of Public Health, University of California Berkeley, Berkeley, CA USA; 7 Faculty of Health Sciences, University of Buea, Buea, Cameroon; 8 Department of Surgery, Soroti Regional Referral Hospital, Soroti, Uganda; 9 Faculty of Health Sciences, University of Bamenda, Bamenda, Cameroon; 10 10833 Le Conte Ave, 72-227 CHS, Los Angeles, CA 90095 USA

**Keywords:** Injury, Physical trauma, Interpersonal violence, Socioeconomic status, Education level, Unemployment, Low- and Middle-Income Countries (LMICs)

## Abstract

**Background:**

Interpersonal violence-related physical injury (IPVRPI) is a leading cause of death in low- and middle-income countries (LMICs), yet reliable data relating socioeconomic status (SES) and IPVRPI in these settings are lacking. We analyzed existing literature on associations between SES and IPVRPI in LMICs to understand how SES is measured in these contexts and synthesize data relating markers of SES to IPVRPI at the individual-level in order to inform future hospital-based IPVRPI prevention efforts.

**Methods:**

We searched Ovid MEDLINE, EMBASE, and Global Health databases in January 2022 for analytical studies from LMICs that explored individual-level associations between IPVRPI and markers of SES. Studies about intimate partner violence, suicide, or children < 12 years old were excluded, as were population-level studies. Markers of SES considered were educational attainment, employment, and household wealth. Collated data relating these SES indicators with IPVRPI were presented in forest plots.

**Results:**

A total of 34 studies from 20 LMICs were included. Brazil, Mexico, and South Africa were the most represented countries. Studies were mostly cross-sectional (*n* = 23), and data were typically from patient hospital records (*n* = 17) or population surveys (*n* = 12). Included studies explored associations between IPVRPI and education (*n* = 26), employment (*n* = 26), and household wealth (*n* = 19). Categorizations, particularly for employment and wealth, were highly variable between studies. Among the studies that performed multivariable analyses, IPVRPI was found to be significantly associated with lower educational attainment (*n* = 6), unemployment (*n* = 4), and lower household wealth (*n* = 6).

**Conclusions:**

Numerous studies have explored individual-level associations between IPVRPI and markers of SES among LMIC populations. Across a variety of LMIC contexts, we found that IPVRPI tended to be associated with markers of lower SES. Further conclusions were limited by the heterogeneity of SES measure categorizations, varied IPVRPI case definitions, and lack of adjusted analyses. Future research should ensure SES measures utilized in LMICs are comprehensive and comparable, focus more specifically on IPVRPI from community violence, and consider hospital-based interventions to reduce risk of IPVRPI in LMIC settings.

**Supplementary Information:**

The online version contains supplementary material available at 10.1186/s12889-025-21321-6.

## Background

Interpersonal violence-related physical injury (IPVRPI), defined as physical injury or death from the intentional use of physical power or force by another person or group, is a leading cause of global mortality [1, 2]. More than 90% of global deaths due to IPVRPI occur in low- and middle-income countries (LMICs) [3], with countries in the Americas and sub-Saharan Africa having the highest homicide rates in the world [1, 2, 4]. In the Americas region in 2019, IPVRPI was the ninth overall cause of death and the leading cause of death for men aged 10–44 years old and women aged 25–34 years old [5]. 

Prevention of interpersonal violence and IPVRPI is a key priority of the World Health Organization [1, 6–11]. Much of the evidence guiding IPVRPI prevention efforts comes from high-income countries (HICs), where IPVRPI is viewed through a public health lens [7, 8, 12–18]. Risk of IPVRPI is known to be multifactorial, influenced by individual, relational, community, and societal factors [1]. Many of these factors are known as social determinants of health (SDH), which serve as “upstream causes or determinants” of health disparities [19–22]. Certain SDH, such as education, employment, income, and wealth, are surrogate markers of an individual’s socioeconomic position or status (SES) and have been targets of interventions to prevent IPVRPI [16, 19, 23–28]. One such intervention that has demonstrated effectiveness in HICs is enrolment in hospital-based violence intervention programs (HVIPs), which aim to reduce risk of injury recidivism after an initial IPVRPI through targeted, individualized interventions [14, 15, 29–33]. HVIPs employ violence prevention professionals to identify patients during their initial hospitalization for IPVRPI and connect them with risk reduction resources and follow up services to address underlying SDH risk factors, with the long-term aim of reducing retaliatory violence and violent injury recidivism [34]. Enrolment in an HVIP has been shown by multiple studies to be associated with reduced IPVRPI recidivism as well as reduced involvement in the criminal justice system [29, 35, 36]. Importantly, most HVIPs focus on IPVRPI inflicted by strangers, acquaintances, and select family members but exclude patients injured as a result of sexual assault, intimate partner violence, and child abuse, as there are often distinct protocols and services in place to deal with those specific patient populations [17, 29, 30, 37, 38]. 

Even though associations between IPVRPI and SES have been well-studied in HIC settings, unique consideration of these associations among LMIC populations is important, due to the distinct social, economic, and political systems in LMICs compared to HICs [7, 8, 12, 39, 40]. Unsurprisingly, availability of data relating measures of SES to IPVRPI among LMICs is sparse [7, 39, 41, 42], with most existing studies exploring these associations at the population level [25, 41, 43–47]. The lack of reliable data relating SES measures to IPVRPI at the individual level limits the ability to develop and implement IPVRPI intervention programs, such as HVIPs, for LMIC populations [9]. Implementation of hospital-based trauma registries in LMICs has helped fill the data gap [48–50]. However, categorizing or measuring SES remains challenging in LMICs, given the prevalence of informal employment, though existing epidemiological studies conducted in LMICs have used asset indices, education, income, and occupation, among other variables [39, 51–55]. 

To strengthen future data collection on IPVRPI and its association with SES in LMICs and to inform future efforts to establish hospital-based interventions to reduce risk of IPVRPI in LMICs, we undertook a systematic review of existing research on individual-level associations between IPVRPI and education, employment, and/or wealth among LMIC populations. The aims of this review were thus to (1) analyze the individual-level markers of SES that have been utilized in recent IPVRPI research conducted in LMICs and (2) collate and synthesize existing data on the individual-level associations between IPVRPI and SES among LMIC populations.

## Methods

### Search strategy

A literature search was conducted through the Ovid MEDLINE, Embase, and Global Health databases to identify articles describing SDH as risk factors for IPVRPI in LMICs. An initial search was conducted in November 2020 and an updated search was performed in January 2022. At the time of the updated search, we focused specifically on associations between IPVRPI and measures of SES, including education, employment, income, and wealth. As such, the search terms “education,” “school,” “university,” “employment,” and “wealth” were added to the search conducted in January 2022. Full search term text is presented in Supplemental Table S1.

### Eligibility criteria

We included original, analytic research studies from LMICs published after 1980 that described individual-level associations between measures of SES and IPVRPI among persons aged 12 years or older. Studies were included if IPVRPI was one of the outcomes. IPRVPI included homicides, non-fatal injuries, or physical pain resulting from the intentional use of physical power or force by another person or group. Studies were excluded if they only examined violent threats or assault without injury, as were studies that focused on suicides or self-inflicted injuries or that grouped self-inflicted injuries with IPVRPI. Additionally, given our desire for this review to inform future HVIP-type efforts in LMICs and in line with the exclusion criteria utilized for HVIPs in HICs, studies that were specifically focused on intimate partner violence, domestic violence, violence against women, sexual violence, rape, child abuse, or elder abuse were excluded [17, 29, 30, 37, 38]. Due to a lack of identified literature that separated partner from non-partner inflicted IPVRPI, we allowed for studies that included partner-perpetrated IPVRPI as a small percentage of the overall study population. To be included, studies also needed to include at least one individual-level SES marker (education, employment, and/or wealth) as an independent predictor of IPRVI. We allowed for measures of wealth (income, asset indices, social class) that were measured at the household-level, rather than individual-level, since this is consistent with established norms in epidemiologic research [39, 56]. Of note, our search also included terms for alcohol use, substance use, and mental health, but articles which focused solely on these risk factors without data on a marker of SES were excluded from the present study. A full list of inclusion and exclusion criteria is presented in Supplemental Table S2.

### Selection process

Search results were imported into EndNote X9 for initial screening and removal of duplicates. Titles were reviewed by a single author (either K.B. or M.D.V.) and studies with relevant titles were uploaded into Covidence [37]. Abstracts were first reviewed independently by two authors (K.B., J.R., M.D.V., or T.T.) for consideration of full text review; disagreements were adjudicated by a third author. Full texts were subsequently reviewed independently for inclusion by two authors (K.B., J.R., M.D.V., T.T., or A.C.), with disagreements adjudicated by a third. The number of included and excluded articles is presented using the preferred reporting items for systematic reviews and meta-analyses (PRISMA) flow diagram [38]. Data extraction was performed by one author (K.B.) and reviewed by a second author (H.T.).

A quality assessment of included studies was performed using select items from the National Institutes of Health (NIH) quality assessment tool for observational cohort and cross-sectional studies [57] and the appraisal tool for cross-sectional studies (AXIS) [58]. The 15 included items had response options of ‘yes,’ ’no,’ or ‘cannot determine.’ After completing all 15 items, each reviewer followed guidance provided by the NIH quality assessment tool instructions to give an overall quality rating for each article [57]. Overall quality rating options included ‘good,’ ‘fair,’ or ‘poor.’ Two authors completed the assessment for each article, with a third author providing a consensus vote as needed. A study’s quality rating was not considered as an exclusion criterion; however, two articles with significant data reliability concerns were excluded based on consensus among authors.

### Synthesis methods

Study characteristics are presented for all included studies, including study country, study type, data source, setting, sex assigned at birth, age of the study population, overall and IPVRPI sample size, percentage of IPVRPI cases that were male, case definition used for IPVRPI, and type of control/comparison group used. We also present whether the included study presented data on education, employment, and/or wealth as they relate to IPVRPI. Of note, a few included studies present descriptive data for an explanatory variable, but data are only presented and discussed in this study if analyzed by the IPVRPI outcome.

For ease of presentation, effect measure data and 95% confidence intervals (95%CI) relating the explanatory variables to IPVRPI are presented in forest plot form. Only data from adjusted analyses are presented, though the specific confounders which are adjusted for varied by study no composite effect measures were calculated given incongruence of reported effect measures and heterogenous variable definitions and reference categories. Adjusted odds ratio (aOR) was the most commonly utilized effect measure, however adjusted prevalence ratio (aPR), risk ratio (aRR), incident rate ratio (aIRR), and hazard ratio (aHR) were also used. Separate forest plots are presented for education, unemployment, and wealth. Given the heterogeneity in variable categories between studies, we present data as follows: the lowest versus highest education category, unemployed versus employed category, and lowest versus highest wealth category. If an effect measure needed to be inverted for inclusion in the forest plot, we simply took the inverse of the aOR (^1^/_aOR_).

## Results

**Fig. 1:**
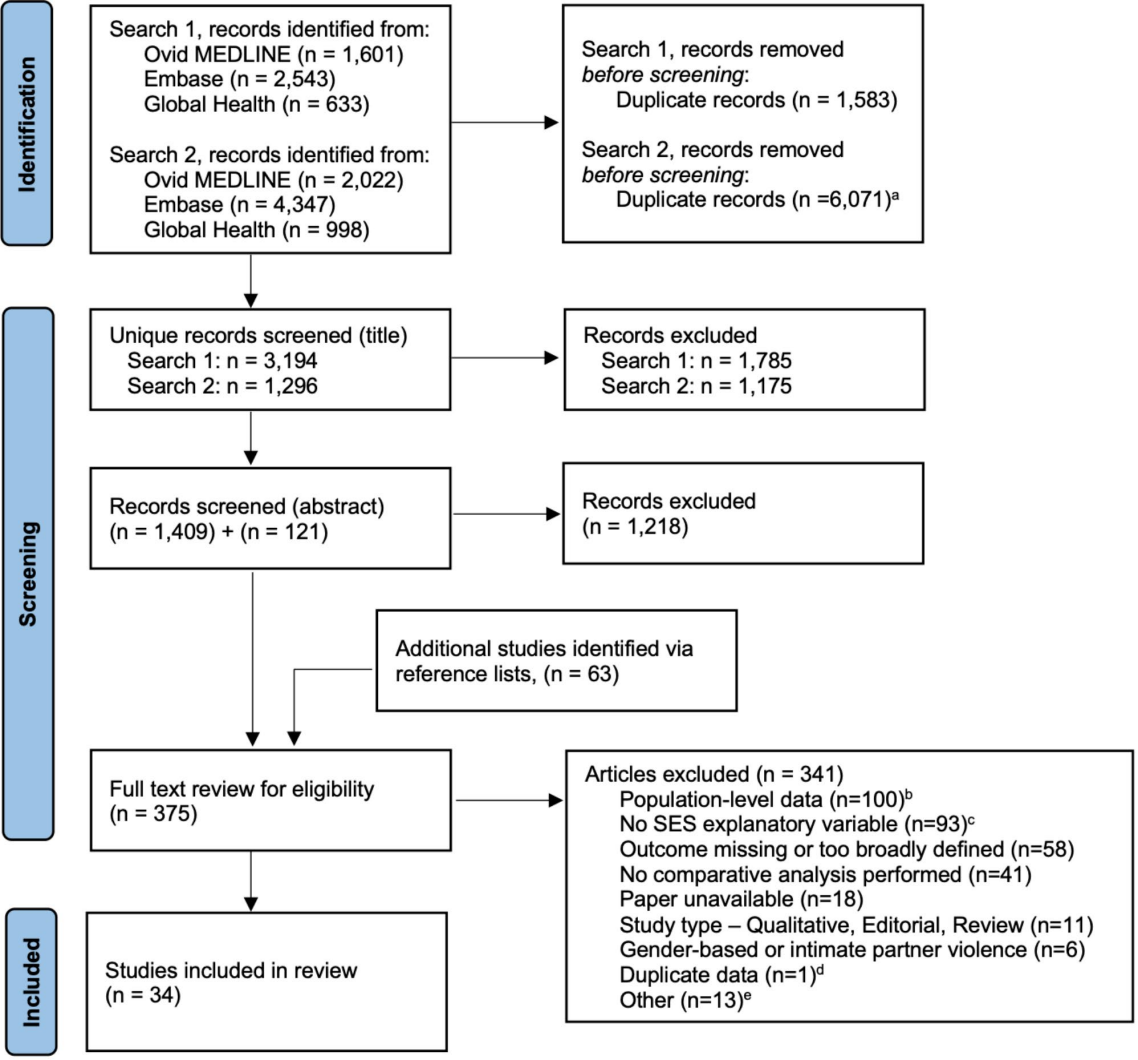
PRISMA flow diagram of included and excluded studies. ^a^Duplicates between the three search databases and duplicates with prior search. ^b^Includes ecological studies, studies with only population-level SES measures, and geospatial analyses. ^c^Majority of these studies (n = 68) explored alcohol or substance use in relation to IPVRPI. ^d^ Two articles - Borges, 1994 [64] and Garcia & Borges, 1991 [93] – present duplicate data. Borges, 1994 was chosen for inclusion since it was published in English. ^e^High income country data (n = 7), outside scope (n = 3), data quality concerns (n = 2), language (n = 1).

After initial exclusions, a total of 375 articles underwent full text review and 34 met inclusion criteria (Fig. 1) (Table 1) [59–92]. Using population-level data, rather than individual-level, was the most common exclusion reason. Included studies were conducted in a total of 20 different LMICs; Brazil (*n* = 4), Mexico (*n* = 4), and South Africa (*n* = 4) were the most common study settings. Study design was most commonly cross-sectional (*n* = 23) or case-control (*n* = 10). IPVRPI case data were obtained from patient hospital records (*n *= 17), population surveys (*n* = 12), or homicide/mortality records (*n* = 5). Among hospital-based studies, nearly all (*n* = 16) used patients with non-violent injuries as the comparison group. Samples sizes varied widely based on study design and setting. Nearly all studies reported a male predominance among the IPVRPI cases. Only six studies presented a breakdown of IPVRPI cases by perpetrator; partner-perpetrated IPVRPI accounted for a minority of the IPVRPI cases, ranging from 3.7 to 26.7%, with lower percentages noted among males compared to females [61, 63, 75, 81, 88, 92]. Results from the quality assessment of included articles are presented in Supplemental Table S3; 20 were rated as good, eight as fair, and six as poor.

**Table 1:** Included studies that explore individual-level associations between markers of socioeconomic status and interpersonal violence-related physical injury among LMIC populations

Author, Year	Country	Type ^b^	Data source, setting	Sex, age range ^g^	Total sample (Cases of IPVRPI ^h^, % cases male ^i^)	IPVRPI case definition and control group	Education categories	Employment categories	Wealth measure and categories
Abdalla, 2014 [59]	Sudan	CS	Survey, household	Both, 0–65+	83,482 (*n* = 84, 74%)	Cases: Respondents with self-reported injury from assault within last 12 months that required any form of healthcare within in the first week Controls: N/A	None Primary Secondary+	-	Wealth index tertiles: Poorest Middle Richest
Bachani, 2017 [60]	Kenya ^a^	CS	Patient records, hospital	Both, 10–24	5,859 (*n* = 1,293, NR)	Cases: Patients with injury from interpersonal violence Controls: Patients with injury from other causes	None or informal Primary school Secondary school Tertiary/college or higher	Office, technical, government Student/not working Casual/daily wage laborer Works in house/homemaker Self-employed Other/unknown	-
Bass, 2018 [61]	The Gambia	CC	Patient records, hospital	Males, 15–45+	894 (*n* = 447, 100%)	Cases: Patients who sought medical treatment for injury from physical violence Controls: Patients who sought treatment for injury from non-violent causes, matched by facility, date, & age	No formal education Primary and secondary Tertiary (college/university)	Civil servant Unemployed Business Student Other	Monthly household income: * ≥*GMD14,000 <GMD14,000
Bass, 2019 [62]	The Gambia	CC	Patient records, hospital	Males, 15–45+	771 (*n* = 257, 100%)	Cases: Patients who presented with injury from physical violence at least twice over the 12-month period Controls: Patients with injury from non-violent causes, matched by facility, date, & age	Primary/no education Secondary education Tertiary education	Employed full time Intermittently employed Unemployed Student	Monthly household income: <GMD15,000 * ≥*GMD15,000
Blair, 2022 [63]	Cameroon	CS	Patient records, hospital	Both, 15–55+	7,605 (*n* = 1,366, 77.6%)	Cases: Patients with intentional injury due to assault, homicide, or legal intervention Controls: Patients with unintentional injury	Primary or no formal Secondary/high school University	Employed Unemployed Retired or housewife Student	Household SES clusters: Rural Urban poor Urban MC homeowner Urban MC tenant Urban wealthy
Borges, 1994 [64]	Mexico	CC	Patient records, hospital	Both, 15–98	400 (*n* = 110, 79.1%)	Cases: Patients with injury from assaults and fights, including those occurring at home Controls: Patients with non-violent injury from animal bites, recreational accidents, or workplace accidents	Elementary or less Secondary or more	White collar Blue collar Student Housewife Other	-
Borges, 1998 [65]	Mexico	CS	Patient records, hospital	Males, 15–99	765 (*n* = 445, 100%)	Cases: Patients with violent injury from fight or an assault Controls: Patients with non-violent injury (workplace, animal bites, recreational)	Incomplete primary Primary-high school Beyond high school	Paid employment Unemployed Student	-
Borges, 2004 [66]	Mexico	CC	Patient records, hospital (Cases) Survey, household (Controls)	Both, 18–60+	1,047 (*n* = 127, 78.0%)	Cases: Patients with injury in which violence was involved Controls: Residents aged 18-65yrs from Pachuca, Mexico	No formal education Elementary Middle High School College or +	Blue collar Farmer Student/part time House keeper Other White collar	-
Cruz, 2014 [67]	Mozambique	CS	Survey, school	Females, 15–45	668 (25.7%, 0%)	Cases: Respondents with minor or severe injury from physical assault by a non-intimate partner in lifetime Controls: N/A	-	Blue collar Low white-collar Middle/high white collar	-
Doolan, 2007 [68]	South Africa	CS	Survey, household	Both, 0–60+	52,906 (*n* = 88, 65.3%)	Cases: Individuals with intentional injury that resulted in treatment by a doctor or nurse in the past 30 days Controls: N/A	Number of years completed	Employed (binary)	Asset index quintiles: Poorest quintile 2nd poorest quintile Middle quintile 2nd richest quintile Richest quintile
Duque, 2011 [69]	Colombia	CS	Survey, household	Both, 12–60	2,095 (7.6%, NR)	Cases: Individuals who were victims of armed physical aggression (injured with sharp object or knife, or shot at with firearm) in their lifetime Controls: N/A	Elementary High school Technical school University or graduate studies	0 months unemployed 1–3 months unemployed 4–8 months unemployed 9–12 months unemployed	Socioeconomic status: Low Middle High
Falbo, 2001 [70]	Brazil	CC	Homicide records (Cases) Survey, household (Controls)	Both, < 20 ^c^	510 (*n* = 255, 95%)	Cases: Homicide victims during the study year (1997) with known identity Controls: Individuals matched by age, sex, and neighborhood, identified within one week of each case	Less than primary school Primary school Secondary school	Unemployment (binary)	Monthly family income: > US $100 * ≤*US $100
Fang, 2014 [71]	China	CS	Survey, household	Both, < 18 ^d^	98,385 (*n* = 101, NR)	Cases: Household member with injury from assault requiring them to miss one day of school or work (non-hospitalized) in previous 12 months Controls: N/A	-	-	Wealth quintiles: Poorest Poorer Middle Richer Richest
Gathecha, 2018 [72]	Kenya	CS	Survey, household	Both, 18–69	4,484 (*n* = 166, 64.6%)	Cases: Individuals seriously injured in a violent incident within the preceding 12 months that required medical attention Controls: N/A	No formal education Primary education Secondary and above	Unemployed Employed Student Homemaker	Wealth quintiles: Poorest Second Middle Fourth Richest
Kelly, 2019 [73]	Liberia	CS	Survey, household	Females, 15–49	4,457 (8.4%, 0%)	Cases: Respondents reporting any non-partner physical violence (hit, slapped, kicked, or anything else to hurt them physically) in the past year Controls: N/A	No education Primary Secondary and above	Worked in the past 12 months Didn’t work in past 12 months	Wealth index: Poorest Poorer Middle Richer Richest
Kibusi, 2013 [74]	Tanzania	CC	Homicide records (Cases) Survey, household (Controls)	Both, All ^e^	301 (*n* = 90, 92.2%)	Cases: Random sample of homicide victims in Dar es Salaam Controls: Population sample matched by sex and age (+/- 5yrs), two controls per case	No education/did not complete elementary school Elementary school completed Secondary school completed/university	Unemployed/retired Unskilled labour Professional/skilled labour Others	-
Leeper, 2019 [75]	South Africa	CS	Patient records, hospital	Both, 14–24	513 (*n* = 324, 80%)	Cases: Assault injured youth presenting for care, excluding sexual assault, child abuse, self-harm Controls: Non-assault injured youth (medical complaint or unintentional injury) matched for gender	-	Currently employed (binary)	Household does not have enough money for things like food & clothes (binary)
Macdonald, 2005 [76]	Mexico & Argentina	CS	Patient records, hospital	Both, 18 + ^f^	2,587 (Mex.) (*n* = 655, 80.5%) 351 (Arg.) (*n* = 55, 69.8%)	Cases: Patients with violent injury, defined as violence being involved in their injury regardless of the type of injury experienced Controls: Patients with accidental injury that did not involve violence	No university (binary)	Unemployed (binary)	Annual family income less than US $10,000 (binary)
Marchese, 2008 [77]	Brazil	CS	Patient records, hospital	Both, 0–60+	583 (*n* = 16, 81.3%)	Cases: Patients with injury due to acts of aggression Controls: Patients with injury from road traffic accidents, falls, or other accidents	*≤ *Elementary > Elementary	-	-
Melo, 2019 [78]	Brazil	CC	Patient records, hospital	Males, 15–29	2,433 (*n* = 811, 100%)	Cases: Patients presenting with injury after assault by unknown assailants Controls: Patients with accidental injury (transport accidents, falls, burns, other accidents)	0–4 years 5–8 years >=8 years	Paid activity (binary)	-
Mian, 2002 [79]	Pakistan	CC	Mortality records (cases) Survey, household (controls)	Both, 16–60	120 (*n* = 35, 97%)	Cases: Individuals killed in Orangi between 1994–1997 due to intentional violence, by firearms, sharp or blunt trauma Controls: Respondents from Orangi with no family member meeting case definition, matched by sex	Average years of education	Unskilled labourers Tailors/embroiderers Weavers Other Unemployed No response	Average monthly income
Orellana, 2017 [80]	Brazil	CS	Mortality records	Both, 15–60+	1,657 (*n* = 913, 93.9%)	Cases: Deaths due to homicide, as defined by ICD-10 codes X85 to Y09 Controls: Deaths due to other external causes during same time period	0–3 years 4–7 years 8–11 years *≥* 12 years	-	-
Osaghae, 2020 [81]	Bangladesh	CS	Survey, household	Both, 11–19	213,782 (*n* = 457, 75.7%)	Cases: Respondents reporting injury from physical violence, defined as injury inflicted directly by another person or from collateral impact, resulting in treatment or lost day of work/school in the past six months Controls: N/A	No education Primary Secondary A Levels College Advanced/professional degree	Agriculture Business Skilled labour (professional) Unskilled/domestic Rickshaw/bus (transport worker) Students Retired/unemployed/housewife Not applicable (others)	Socioeconomic index: Lowest Low Middle High Highest
Otieno, 2015 [82]	South Africa	CO	Survey, household	Both, 0–65+	126,462 (*n* = 536, NR)	Cases: Homicide related death, defined as intentional killing by another person or criminal negligence that causes the death of another person Controls: N/A	None Primary Post-primary	-	Socioeconomic status: Poorest Very Poor Poor Less Poor Least Poor
Oyefeso, 2011 [83]	Namibia	CS	Patient records, hospital	Both, 0–70+	331 (*n* = 100, NR)	Cases: Patients with intentional injury due to cuts/stabs, assaults, human bites, gunshots Controls: Patients with unintentional injury (motor vehicle accident, falls, sports, donkey cart accidents, others)	-	School pupil Unemployed Children/infants Farm workers Others	-
Purcell, 2020 [84]	Malawi	CS	Patient records, hospital	Both, 15–45+	87,338 (*n* = 30,523, 80.1%)	Cases: Patients with traumatic injury due to assault Controls: Patients with unintentional injury	-	Unemployed (binary)	-
Rubanzana, 2015 [85]	Rwanda	CC	Mortality records (Cases) Survey, household (Controls)	Both, 18–75	624 (*n* = 156, 57.0%)	Cases: Victims of homicide as determined by the police criminal investigation department Controls: Respondents matched by age (+/- 5yrs), gender, and area of residence	None Primary Secondary or tertiary	Employed Dealing in illegal activities Unemployed/other	-
Salamati, 2015 [86]	Iran	CS	Survey, household	Both, 15–64	7,886 (*n* = 24, 66.7%)	Cases: Interpersonal violence related physical injury during the previous three months Controls: N/A	Years of full-time education	Employed (binary)	-
Tadesse, 2014 [87]	Ethiopia	CS	Patient records, hospital	Both, < 20–60+	321 (*n* = 72, 90.3%)	Cases: Patients with intentional injury from assault Controls: Patients with unintentional injury	-	-	Monthly income: <650ETB * ≥*650ETB
Tadesse, 2015 [88]	Ethiopia	CS	Patient records, hospital	Both, < 20–60+	379 (*n* = 108, 85.2%)	Cases: Patients with interpersonal violence related injury Controls: Patients with injury due to other mechanisms	-	-	Low monthly family income (binary)
van der Westhuizen, 2017 [89]	South Africa	CS	Patient records, hospital	Both, 18–40+	200 (*n* = 118, 78.0%)	Cases: Patients presenting for treatment of injury due to assault Controls: Patients presenting for treatment of injury from unintentional causes	Completed high school (binary)	Employed (binary)	-
Yang, 2016 [90]	China	CS	Survey, household	Females, 15–79	8,071 (10.7%, 0%)	Cases: Respondents reporting injury in the past year inflicted by another person in a deliberate attack that required medical care or rest for minimum ½ day Controls: N/A	Junior high school or less High school Junior college or college	Managers and clerks Professionals Commerce and service Operations Students Retired Other	-
Yang, 2020 [91]	China	CS	Survey, school	Both, 18–23	4,903 (4.7%, NR)	Cases: Respondent reporting any violent injury during the past 12 months that required medical care or rest for minimum ½ day Controls: N/A	-	-	Past year family income: <¥10,000 ¥10,000–19,999 * ≥*¥20,000
Yu, 2020 [92]	The Gambia	CC	Patient records, hospital	Females, 15–45+	388 (*n* = 194, 0%)	Cases: Patients who sought treatment for injury from physical violence Controls: Patients with injury due to traffic crashes, falls, sports, and other nonviolent causes matched by facility, date, sex, and age	No formal education Primary and secondary Tertiary (college/university)	Employed Unemployed Housewife Student	Monthly household income < GMD 14,000 (binary)

^a^ Study also presents data from Oman, but no data for the markers of SES are presented in relation to IPVRI

^b^ Study types include cross-sectional (CS), cohort (CO), and case-control (CC)

^c^ Large majority (93%) of sample were between 15-19yrs old, so the study team allowed for inclusion

^d^ The majority of assaults occurred in those age 10–17, so the study team allowed for inclusion

^e^ No age range is provided. Mean (SD) age was 32.4 (10.8) for cases and 32.1 (10.4) for controls

^f^ No upper limit of age range is provided. Among the violent injury group, 63.1% (Mexico) and 62.3% (Argentina) were between the ages of 18 and 29 years old

^g^ Presentation of age summary data varied by publication, with most studies only presenting categorized age data. Despite the variability, all included studies had a mean or median age at or below 44 years old

^h^ Most studies present IPVRPI sample size (n). Five studies only present the prevalence (%) of IPVRPI cases

^i^ The percentage of each study’s IPVRPI sample occurring in males is reported when available. Otherwise labeled as not reported (NR)

### Education

**Fig. 2:**
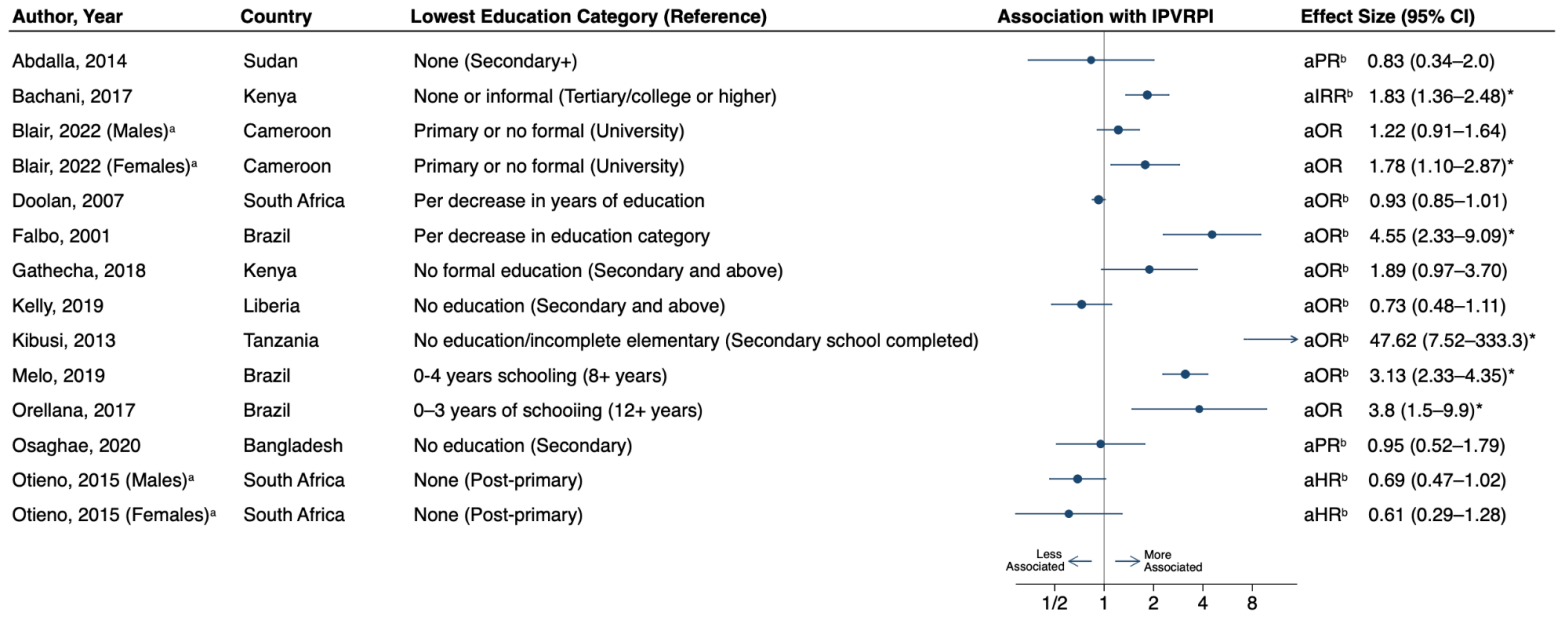
Forest plot of associations between lowest education category and interpersonal violence-related physical injury among individuals from low- and middle-income countries. ^a^Studies with two rows of data due to results stratified by sex. ^b^Effect measure and confidence interval inverted. ^*^ Significant association

The association between educational attainment and IPVRPI was explored by a majority of the included studies (*n* = 26). Most utilized a categorized education level with some combination of no formal, primary-level, secondary-level, and tertiary-level education categories, though the reference groups used in regression analyses was variable. Three studies utilized a quantitative variable of number of years of education completed [68, 79, 86]. Among the 26 studies considering education, 12 had data amenable to inclusion in a forest plot (Fig. 2). Six studies found lower education to be significantly associated with IPVRPI [60, 63, 70, 74, 78, 80].

### Unemployment

**Fig. 3:**
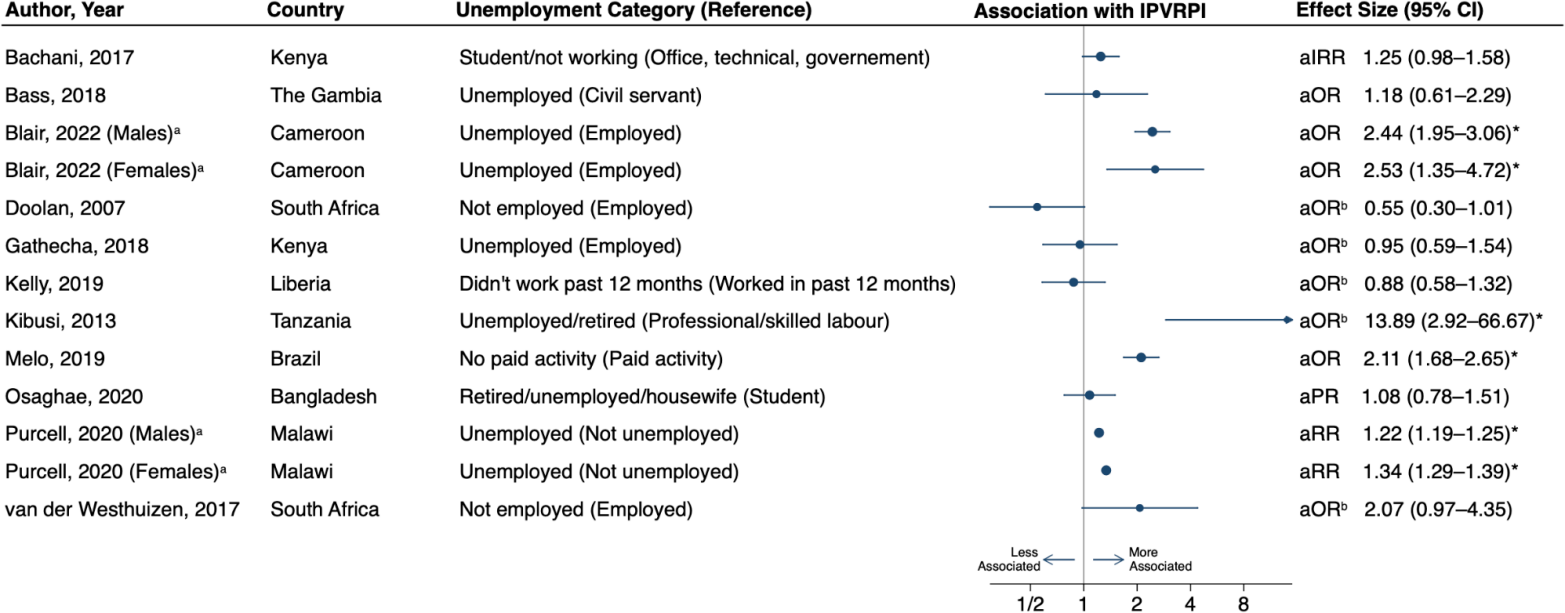
Forest plot of associations between unemployment and interpersonal violence-related physical injury among individuals from low- and middle-income countries. ^a^Studies with two rows of data due to results stratified by sex. ^b^Effect measure and confidence interval inverted. ^*^Significant association

There were 26 studies that considered employment or occupation in relation to IPVRPI, with highly variable categorizations. Nine studies used a binary categorization for work or employment [68, 70, 73, 75, 76, 78, 84, 86, 89], and one additional study included time qualifiers for unemployment (how many months of the past year) [69]. Six studies had additional categories alongside employed or unemployed, such as student or housewife [62, 63, 65, 72, 85, 92]. The remaining ten studies specified certain occupation or job types, rather than a composite “employed” category; [60, 61, 64, 66, 67, 74, 79, 81, 83, 90] of those, six had a category for unemployed [60, 61, 74, 79, 81, 83]. In total, there were 11 studies with an unemployed category that performed multivariable analyses and were amenable to inclusion in a forest plot (Fig. 3). Of those, unemployment had a significant positive association with IPVRPI in four studies [63, 74, 78, 84].

### Household wealth

**Fig. 4:**
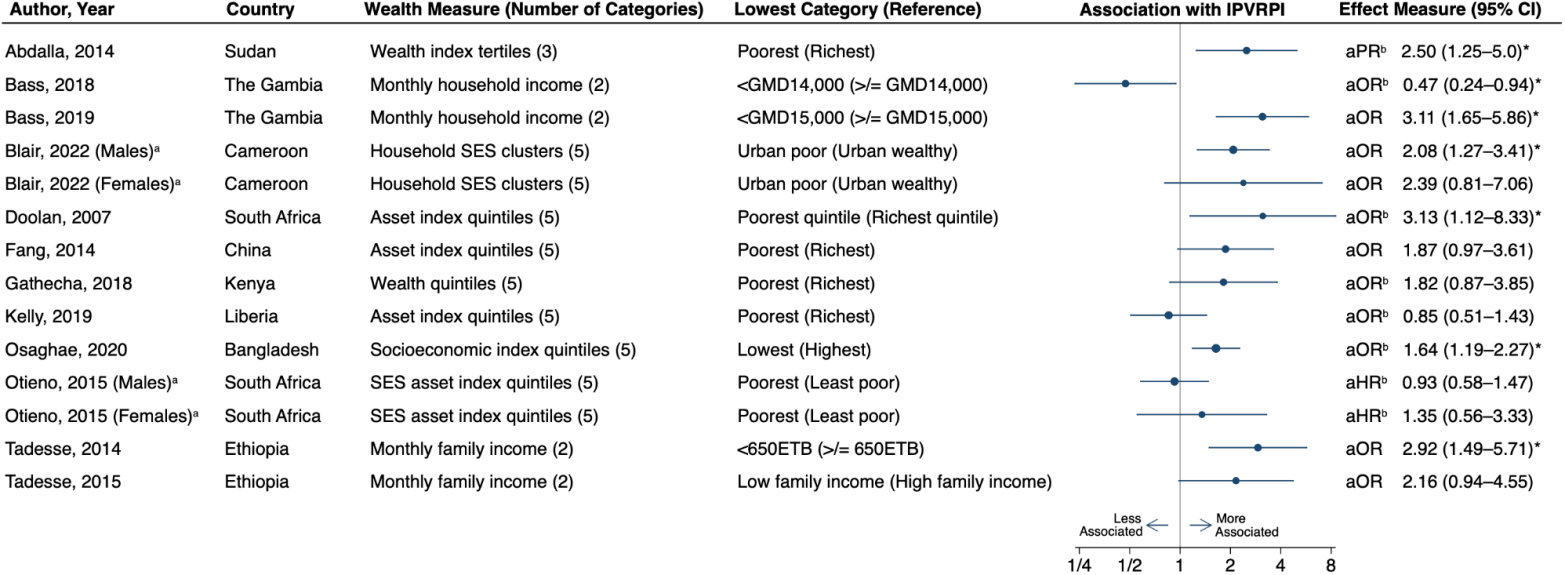
Forest plot of associations between the lowest household wealth category and interpersonal violence-related physical injury among individuals from low- and middle-income countries. ^a^Studies with two rows of data due to results stratified by sex. ^b^Effect measure and confidence interval inverted. ^*^Significant association

There were 19 studies which explored the association between household wealth and IPVRPI. Most of these used asset indices or SES groupings to categorize households into tertiles [59, 69], quintiles [68, 71–73, 81, 82], or economic clusters [63]. Others utilized household monthly [61, 62, 70, 79, 87, 88, 92], or annual income [76, 91]. One publications assessed whether the household had enough money for necessities [75]. Of the 12 studies with data amenable to inclusion in a forest plot, half (*n* = 6) found a significant association between IPVRPI and being from a lower wealth household (Fig. 4) [59, 62, 63, 68, 81, 87]. Conversely, only one found significant associations between IPVRPI and higher household wealth [61].

## Discussion

Thirty-four studies explored individual-level associations between SES indicators and IPVRPI among LMIC populations. These analyses predominantly used categorical SES measures, with highly variable categorizations, particularly for employment and wealth. Despite the heterogeneity of categorizations, lower educational attainment, unemployment, and lower wealth were consistently shown to be associated with IPVRPI. Among the studies that performed adjusted analyses, IPVRPI was shown to be significantly associated with markers of lower SES across continents and study settings.

### Education

In line with our findings, population-level analyses have shown homicide rates tend to be higher in areas or among communities with lower education attainment [25, 44, 45, 94]. Education may protect against IPVRPI at the individual-level in many ways; the degree or diploma, skills attained, time engaged in school over other activities, and interpersonal school-based relationships may all contribute [16, 42]. However, the influence of education on IPVRPI is complex and interrelated with other SES factors, such as employment, as well as demographic characteristics [16, 44]. Education-related initiatives to reduce IPVRPI are often discussed, such as incentives for completion of secondary school or equivalence degrees [16, 23, 26], but there remains a dearth of evidence from LMICs on how these initiatives may impact IPVRPI [13, 42]. A study of violently injured patients enrolled in an HVIP in the United States showed that nearly two thirds identified furthering education as a priority on their initial needs assessment, and that meeting that need was associated with reduced risk of IPVRPI recidivism [30, 95]. The theory of change for HVIPs suggests that education facilitates employment, self-determination, and economic stability, all of which contribute to reduced risk of injury recidivism [29, 32, 96]. 

Among the measures of SES considered in this review, measurement of education was seemingly the most consistent, however the number and completion of education levels as well as the reference category used varied greatly. Some studies defined educational attainment by completion of an educational level, whereas others categorized based solely on initiation of that level. The Demographic and Health Survey (DHS), which was the data source for two of the studies in this review [68, 73], specifies whether a level was completed. The number of years that comprised an educational level also varied between studies, and a few included education categories unique to the country that may not be easily comparable to other settings, such as technical school in Colombia [69] or A-levels in Bangledesh [81]. Given education is frequently included as an explanatory variable in epidemiological studies, the International Stratification and Mobility File (ISMF) has sought to harmonize its measurement across countries to improve comparability [97]. A cutoff of six years of education for complete primary and 12 years for complete secondary is commonly used [97]. Aside from level of education, some studies looking at IPVRPI also considered school-type, such as whether a school was private versus public [98–100], or the level of university [91]. Others used parental or head of household educational attainment as a surrogate measure of SES [39, 68, 70, 88], with higher parental education levels demonstrating a potential protective effect against an individual’s risk of IPVRPI [70, 88]. 

There are important contextual considerations when interpreting education level in LMICs. Increased educational attainment may not always correlate with increased socioeconomic position; for example, in certain settings, older adults may have high social standing but limited formal education [51]. An individual’s sex at birth may influence access to education in certain LMIC contexts [39], which is an important confounder to consider when studying associations with IPVRPI, the prevalence of which is much higher in males than females [1]. Given this and other sex differences with regards to IPVRPI in LMICs, we think that analyses should be stratified by sex when possible. Among the few studies that evaluated the association between education and IPVRPI among females specifically, results were mixed. Lower educational attainment was associated with significantly increased odds of IPVRPI among Cameroonian women [63], but the converse was true among Chinese women [90]. 

### Employment

Individual- and population-level unemployment is well accepted as a risk factor for violence and IPVRPI, though the relationship is complex and there remains a lack of evidence to demonstrate causality [1, 11, 16, 101, 102]. Several studies included in this review found unemployment to be significantly associated with IPVRPI, but heterogenous categorization of employment and occupation limited our ability to draw further conclusions. Although we did not consider employment of other household members or neighborhood-level unemployment, both can be used as surrogate measures of SES, and multiple studies have shown protection against IPRVI [68, 70, 90, 91]. For the individual, employment is thought to reduce risk of IPVRPI through economic opportunity, individual agency, social connectedness, community participation, learned professional behavior, and reduction in free time [16, 103]. As mentioned above, the theory of change model for HVIPs considers education and employment to be interrelated, both of which contribute to an individual being able to meet their needs and to have economic stability [96]. Among patients enrolled in an HVIP after experiencing an IPVRPI, obtaining employment is commonly identified as a need [95]. Numerous studies have shown involvement in HVIPs to lead to increased employment, though causality with reduced IPVRPI recidivism has not been demonstrated [29, 35]. 

Consistent and appropriate definitions of “unemployed” should be a priority for future research. Many studies grouped the categories of “housewife”, “retired,” or “student” within “unemployed,” but these categories likely portend lower risk of IPVRPI than true unemployment, and their grouping with unemployed may mask an association with IPVRPI [63]. Ideally, the category of unemployed should only include those who are eligible and able to work, but currently not employed [84], though separating those who are unable to work may be limited by sample size [63]. The DHS defines current work or employment as having worked in the last seven days, but also asks about work in the prior 12 months [68, 73]. Some studies differentiated between part-time and full-time employment, seasonal work, and duration of unemployment in the prior year, which is commendable if there is a sufficient sample size. Consideration of informal or seasonal work is of particular importance in LMIC settings [39]. For studies that included youth, distinguishing between student and employment can be difficult. Some studies used separate questions for employment and school attendance [70, 75], while others include student as an occupation or employment category [60, 63, 81], which fails to capture students who work part-time.

There were ten studies included in this review that listed specific occupations, but the categorizations were highly variable and poorly defined, which limits the ability for those data to inform policy or interventions. Having a “blue-collar” compared to “white collar” occupation was associated with IPVRPI [66, 67], and men in The Gambia who worked in business had higher adjusted odds of IPVRPI when compared to those who worked in civil service [61]. The International Standard Classification for Occupations (ISCO) provides the following ten groupings: managers; professionals; technicians and associate professionals; clerical support workers; service and sales workers; skilled agricultural, forestry, and fishery workers; craft and related trades workers; plant and machine operators and assemblers; elementary occupations; and armed forces occupations [104]. These categorizations are used by the DHS, and our study group has incorporated them into our trauma registry collection form in Cameroon.

### Wealth

Among the studies that performed adjusted analyses, half found lower household wealth to be significantly associated with IPVRPI. This is consistent with population-level data, which have shown homicide rates to be inversely related to mean family income and per-capita GDP [41, 43, 94]. The relationship between poverty and IPVRPI is likely related to resource insecurity, lack of opportunities, and the need for protection, which notably also contribute to recruitment into gangs or illegal activity in impoverished areas [16]. Many argue that it is not poverty in and of itself that portends higher risk of IPVRPI, but rather the presence of poverty alongside high levels of income inequality [16, 25, 41, 47, 105]. Income inequality is thought to contribute to IPVRPI via decreased social cohesion, increased stress, and increased hostility, among other things [105]. The HVIP theory of change posits that increased income or wealth affords an individual economic stability and promotes overall wellbeing, which in turn reduces IPVRPI recidivism, though this is difficult to measure in a program evaluation [96]. 

Measuring household wealth in LMICs can be challenging [39, 51, 52]. While we sought to consider individual-level explanatory variables, we found measures of wealth to be consistently collected at the household level. With a focus on individual-level risk of IPVRPI, it is important to consider the individual’s relationship to the household, as their household position will mediate access to assets and material wealth [39]. The appropriate wealth measure varies by degree of the household’s participation in the formal workforce and cash economy, consistency of employment, and more [39]. Several studies utilized monthly or annual household income as a measure of wealth, but income can be an incomplete metric of wealth in LMICs, as it fails to capture informal economy participation, self-employment, and seasonal jobs [39, 51]. For example, the 2018 Cameroon DHS found that 35.5% of men and 36.9% of women were paid at least partially by methods other than cash [106]. The use of income also fails to capture extended family and community support, inherited assets, etc.

Given the limitations of using income, many epidemiological surveys, including the DHS, utilize wealth or asset indices to capture a household’s SES, which attempt to quantify a household’s resources relative to other households in the country or community [39, 51, 53, 107]. Wealth indices are generally accepted as more accurate than income measures for SES assessments among LMIC populations, though limitations still exist [53]. The length of most asset index questionnaires limits their widespread use and may be particularly impractical for trauma registry data collection and hospital-based studies of IPVRPI. For this reason, Eyler et al. (2016) developed a condensed asset-based *EconomicClusters* model that only requires data from a few household asset questions [52, 108]. An additional limitation of wealth indices is that certain assets carry different socioeconomic significance in different settings, and that wealth indices still incompletely capture the multi-dimensional relationship between prosperity and health [53]. Although it was outside our scope, area-level measures of SES are also commonly used in epidemiologic studies; [109] increased area-level disadvantage has been shown to be associated with IPVRPI in Mexico and South Africa [110, 111]. 

### Limitations and recommendations for further research

There are some important limitations to this systematic review. As a review of observational studies, we describe associations with IPVRPI but cannot assess causation. The studies included in this review were published over a nearly 30-year period, from 1994 to 2022, during which time there has been significant shifts in global patterns of violence and IPVRPI. While most studies were published in the past decade, there remains a need for additional high-quality, up to date data on the relationship between IPVRPI and SES in LMIC settings.

Varied definitions of IPVRPI, particularly among the population survey-based studies, and reliance on self-report to classify injury mechanism and intent raise the possibility of misclassification and recall bias. Studies at highest risk of case misclassification due to broad definitions of IPVRPI were excluded. Our intent was to focus this review on the population of violently injured individuals in LMICs that would be considered for individual-level, targeted interventions such as HVIPs. To that end, we excluded studies exclusively focused on sexual assault and intimate partner violence, as mentioned above. However, given the dearth of literature on IPVRPI in relation to individual-level SES in LMICs, we accepted that nearly all of the studies in this review included a small percentage of partner-perpetrated IPVRPI within their overall study population. Of note, most studies had a strong male predominance among IPVRPI cases, and the percentage of partner-perpetrated IPVRPI was shown to be lower among males than females. This lack of study population specificity is reflective of an area of research that is in its early stages, and there remains a need for future work that only considers those with IPVRPI from community violence, which would exclude those injured by partners or family. Lastly, this study makes frequent mention of the HVIP model and its effectiveness in reducing IPVRPI recidivism in HIC settings, but the feasibility of such a resource intensive intervention in an LMIC setting is another important area of future research.

The high level of variability in SES variable categories limited our ability to conduct a meta-analysis, but our review of these categories has allowed us to make several recommendations to ensure future analyses of SES and IPVRPI in LMICs are accurate, replicable, and comparable across contexts. Measurement of an individual’s education level should utilize the definitions provided by the ISMF and should include whether a level of education was complete or incomplete [97]. Current employment should be defined as work in the previous seven days, with additional consideration of work in the previous year [68, 73]. Occupation categories should align with the ISCO occupational groupings, and unemployed should be a categorization distinct from retired, homemaker/housewife, or student [104]. Lastly, we feel that household wealth should be measured using asset indices, including in the hospital-based study setting [52, 108]. 

This study is limited to individual and household-level SES factors, but area-level risk factors for IPVRPI are also important to consider in future research. An individual’s environment, which can be described through a breadth of variables, including area-level deprivation, alcohol outlet density, and environmental markers of social cohesion such as parks and recreation facilities, is an important contributor to crime and interpersonal violence [110, 111]. Increased individual-level socioeconomic position cannot fully mitigate negative environmental factors that contribute to IPVRPI [51]. Additionally, there are a number of other SDH that are related to SES that have been considered in relation to IPVRPI in LMICs that were not included in this review.

As mentioned above, measuring SES in LMICs is challenging. These findings must be interpreted with the understanding that the use of education, employment, income, or asset indices all have limitations and fail to fully capture the multidimensional nature of wealth and prosperity and the relationship with health outcomes. Within countries, there may be many different paths to prosperity and improved health that vary between urban and rural areas and depend upon the level of involvement in the cash economy [39, 53]. Between countries, different SES measures may have different implications [53]. Additionally, we discuss the results above assuming a unidimensional, linear association between SES and IPVRPI, where risk of IPVRPI decreases as SES increases, but the articles reviewed did not always demonstrate a linear association, suggesting that the relationship between SES and IPVRPI is more complex. Lastly, there is likely a bi-directional association with SES that was not discussed here, in which ill-health, including IPVRPI, limits educational and economic opportunities. This may be particularly true for younger age groups and in low-income countries where worker protections are sparse [39]. 

## Conclusion

This systematic review offers evidence from a variety of LMIC contexts and populations that IPVRPI tends to be associated with markers of lower, rather than higher, SES. Our analysis also highlights the challenge of capturing an individual’s SES as it relates to IPVRPI, especially in LMIC settings. The heterogeneity of categories and reference levels used for markers of SES in LMICs make generalizability of findings and comparisons across contexts difficult. Future research should carefully consider how to measure education, employment, and wealth to ensure the assessment of SES as it relates to IPVRPI or other health conditions is accurate, replicable, and comparable across contexts. Ultimately, the goal of this work is to inform future prospective studies on SES as it relates to IPVRPI from community violence in LMICs, and to provide a foundation for hospital-based interventions to reduce the risk of IPVRPI globally, particularly for individuals of lower socioeconomic position.

## Electronic supplementary material

Below is the link to the electronic supplementary material.

Supplementary Material 1

## Data Availability

No datasets were generated or analysed during the current study.

## Abbreviations

95%CI: 95% Confidence Interval
aHR: Adjusted Hazard Ratio
aIRR: Adjusted Incident Rate Ratio
aOR: Adjusted Odds Ratio
aPR: Adjusted Prevalence Ratio
aRR: Adjusted Risk Ratio
AXIS: Appraisal Tool for Cross-Sectional Studies
CC: Case control study
CO: Cohort study
CS: Cross-sectional study
DHS: Demographic and Health Survey
HIC: High-income country
ISCO: International Standard Classification for Occupations
IPVRPI: Interpersonal violence-related physical injury
ISMF: International Stratification and Mobility File
LMIC: Low- and middle-income country
MC: Middle class
N/A: Not applicable
NIH: National Institutes of Health
NR: Not Reported
PRISMA: Preferred Reporting Items for Systematic Reviews and Meta-Analyses
SES: Socioeconomic status
SDH: Social determinant of health
