# Supplementary material for: Interpersonal violence-related physical injury in low- and middle-income countries and its association with markers of socioeconomic status: a systematic review
Source: BMC Public Health. 2025 Mar 19;25:1065. doi: 10.1186/s12889-025-21321-6 (PMC11924722; doi:10.1186/s12889-025-21321-6)
Supplement: Supplementary file 1 — Supplementary Material 1 [file 12889_2025_21321_MOESM1_ESM.pdf]

Supplemental Table S1: Systematic review database search terms [a]

Database	Search Terms
Ovid MEDLINE	("Gun Violence"/ or "Homicide"/ or ("violent injur*" or homicide* or "intentional injur*" or murder* or "interpersonal violence" or assault* or "firearm injur*" or "gun violence" or "gang violence" or "penetrating trauma").ti,ab,kw.) and (exp Risk Factors/ OR exp Socioeconomic Factors/ OR exp mental disorders/ OR exp Poverty/ OR exp unemployment/ OR exp **Employment**/ OR exp **Education**/ or (risk* or socioeconomic or "social determinant*" or "mental illness*" or poverty or alcohol* or unemploy* or **employ*** or **school** or **university or wealth**).ti,ab,kw or ((substance or drug or drugs) adj2 ("use" or abuse or misuse)).ti,ab,kw) and (exp "Developing Countries"/ or LMIC.ti,ab,kw. or (("low income" or "middle income" or "low and middle income" or "low or middle income") adj1 (country or countries)).ti,ab,kw. or (exp "Africa"/ or "Latin America"/ or "Central America"/ or "Caribbean Region"/ or "South America"/ or "Mexico"/ or "Asia, Central"/ or "Asia, Northern"/ or "Borneo"/ or "Brunei"/ or "Cambodia"/ or "Indochina"/ or "Indonesia"/ or "Laos"/ or "Malaysia"/ or "Mekong Valley"/ or "Myanmar"/ or "Philippines"/ or "Thailand"/ or "Timor-Leste"/ or "Vietnam"/ or "Asia, Western"/ or "Mongolia"/ or "Albania"/ or "Baltic States"/ or "Bosnia and Herzegovina"/ or "Bulgaria"/ or "Hungary"/ or "Kosovo"/ or "Montenegro"/ or "Republic of Belarus"/ or "Republic of North Macedonia"/ or "Serbia"/ or "Ukraine"/ or "Transcaucasia"/ or (Afghanistan or Angola or Albania or Argentina or Armenia or "American Samoa" or Azerbaijan or Burundi or Benin or Burkina Faso or Bangladesh or Bulgaria or Bosnia or Herzegovina or Belarus or Belize or Bolivia or Brazil or Bhutan or Botswana or "Central African Republic" or China or "Ivory Coast" or Ivoire or Cameroon or Congo or Colombia or Comoros or "Cabo Verde" or "Costa Rica" or Cuba or Djibouti or Dominica or "Dominican Republic" or Algeria or Ecuador or Egypt or Eritrea or Ethiopia or Fiji or Micronesia or Gabon or Georgia or Ghana or Guinea or Gambia or Grenada or Guatemala or Guyana or Honduras or Haiti or Indonesia or India or Iran or Iraq or Jamaica or Jordan or Kazakhstan or Kenya or Kyrgyz* or Cambodia or Kiribati or Lao or Laos or Lebanon or Liberia or Libya or Lucia or "Sri Lanka" or Lesotho or Morocco or Moldova or Madagascar or Maldives or Mexico or "Marshall Islands" or Macedonia or Mali or Myanmar or Montenegro or Mongolia or Mozambique or Mauritania or Malawi or Malaysia or Namibia or Niger or Nigeria or Nicaragua or Nepal or Pakistan or Peru or Philippines or "Papua New Guinea" or "North Korea" or DPRK or Paraguay or "West Bank" or Gaza or Russia* or Rwanda or Sudan or Senegal or "Solomon Islands" or "Sierra Leone" or "El Salvador" or Somalia or Serbia or "Sao Tome" or Principe or Suriname or Eswatini or Syria* or Chad or Togo or Thailand or Tajikistan or Turkmenistan or Timor or Tonga or Tunisia or Turkey or Tuvalu or Tanzania or Uganda or Ukraine or Uzbekistan or Grenadines or Venezuela or Vietnam or Vanuatu or Samoa or Kosovo or Yemen or "South Africa" or Zambia or Zimbabwe).ti,ab,kw.))
Embase	('gun violence'/exp OR 'gun violence' OR 'homicide'/exp OR 'homicide' OR 'violent injur*':ti,ab,kw OR homicide*:ti,ab,kw OR 'intentional injur*':ti,ab,kw OR murder*:ti,ab,kw OR 'interpersonal violence':ti,ab,kw OR assault*:ti,ab,kw OR 'firearm injur*':ti,ab,kw OR 'gun violence':ti,ab,kw OR 'gang violence':ti,ab,kw OR 'penetrating trauma':ti,ab,kw) AND (('risk factor'/exp OR 'risk factor') AND ('socioeconomics'/exp OR 'socioeconomics') OR 'mental disease'/exp OR 'mental disease' OR 'unemployment'/exp OR **'employment'**/exp OR 'unemployment' OR 'illicit drug'/exp OR 'illicit drug' OR 'social determinants of health'/exp OR 'social determinants of health' OR risk*:ti,ab,kw OR socioeconomic:ti,ab,kw OR 'social determinant*':ti,ab,kw OR 'mental illness*':ti,ab,kw OR poverty:ti,ab,kw OR alcohol*:ti,ab,kw OR **employ*** OR unemploy*:ti,ab,kw OR **'education'**:ti,ab,kw OR **'wealth'**:ti,ab,kw OR **'school'**:ti,ab,kw OR **'university'**:ti,ab,kw OR ((substance:ti,ab,kw OR drug:ti,ab,kw OR drugs:ti,ab,kw) AND adj2:ti,ab,kw AND ('use':ti,ab,kw OR abuse:ti,ab,kw OR misuse:ti,ab,kw))) AND ('developing country'/exp OR 'developing country' OR 'low income country'/exp OR 'low income country' OR 'middle income country'/exp OR 'middle income country' OR lmic:ti,ab,kw OR (('low income':ti,ab,kw OR 'middle income':ti,ab,kw OR 'low and middle income':ti,ab,kw OR 'low or middle income':ti,ab,kw) AND adj1:ti,ab,kw AND (country:ti,ab,kw OR countries:ti,ab,kw)) OR 'africa'/exp OR 'africa' OR 'south and central america'/exp OR 'south and central america' OR 'mexico'/exp OR 'mexico' OR 'kazakhstan'/exp OR 'kazakhstan' OR 'kyrgyzstan'/exp OR 'kyrgyzstan' OR 'iran'/exp OR 'iran' OR 'iraq'/exp OR 'iraq' OR 'jordan'/exp OR 'jordan' OR 'lebanon'/exp OR 'lebanon' OR 'palestine'/exp OR 'palestine' OR 'syrian arab republic'/exp OR 'syrian arab republic' OR 'turkey (republic)'/exp OR 'turkey (republic)' OR 'yemen'/exp OR 'yemen' OR 'south asia'/exp OR 'south asia' OR 'tajikistan'/exp OR 'tajikistan' OR 'turkmenstein' OR 'uzbekistan'/exp OR 'uzbekistan' OR 'china'/exp OR 'china' OR 'north korea'/exp OR 'north korea' OR 'mongolia'/exp OR 'mongolia' OR 'philippines'/exp OR 'philippines' OR 'borneo'/exp OR 'borneo' OR 'brunei darussalam'/exp OR 'brunei darussalam' OR 'cambodia'/exp OR 'cambodia' OR 'indonesia'/exp OR 'indonesia' OR 'laos'/exp OR 'laos' OR 'malaysia'/exp OR 'malaysia' OR 'myanmar'/exp OR 'myanmar' OR 'papua new guinea'/exp OR 'papua new guinea' OR 'thailand'/exp OR 'thailand' OR 'timor-leste'/exp OR 'timor-leste' OR 'viet nam'/exp OR 'viet nam' OR 'albania'/exp OR 'albania' OR 'armenia'/exp OR 'armenia' OR 'azerbaijan'/exp OR 'azerbaijan' OR 'belarus'/exp OR 'belarus' OR 'bosnia and herzegovina'/exp

	OR 'bosnia and herzegovina' OR 'bulgaria'/exp OR 'bulgaria' OR 'georgia'/exp OR 'georgia' OR 'moldova'/exp OR 'moldova' OR 'montenegro'/exp OR 'montenegro' OR 'republic of north macedonia'/exp OR 'republic of north macedonia' OR 'russian federation'/exp OR 'russian federation' OR 'serbia'/exp OR 'serbia' OR 'ukraine'/exp OR 'ukraine' OR afghanistan:ti,ab,kw OR angola:ti,ab,kw OR albania:ti,ab,kw OR argentina:ti,ab,kw OR armenia:ti,ab,kw OR 'american samoa':ti,ab,kw OR azerbaijan:ti,ab,kw OR burundi:ti,ab,kw OR benin:ti,ab,kw OR 'burkina faso':ti,ab,kw OR bangladesh:ti,ab,kw OR bulgaria:ti,ab,kw OR bosnia:ti,ab,kw OR herzegovina:ti,ab,kw OR belarus:ti,ab,kw OR belize:ti,ab,kw OR bolivia:ti,ab,kw OR brazil:ti,ab,kw OR bhutan:ti,ab,kw OR botswana:ti,ab,kw OR 'central african republic':ti,ab,kw OR china:ti,ab,kw OR 'ivory coast':ti,ab,kw OR ivoire:ti,ab,kw OR cameroon:ti,ab,kw OR congo:ti,ab,kw OR colombia:ti,ab,kw OR comoros:ti,ab,kw OR 'cabo verde':ti,ab,kw OR 'costa rica':ti,ab,kw OR cuba:ti,ab,kw OR djibouti:ti,ab,kw OR dominica:ti,ab,kw OR 'dominican republic':ti,ab,kw OR algeria:ti,ab,kw OR ecuador:ti,ab,kw OR egypt:ti,ab,kw OR eritrea:ti,ab,kw OR ethiopia:ti,ab,kw OR fiji:ti,ab,kw OR micronesia:ti,ab,kw OR gabon:ti,ab,kw OR georgia:ti,ab,kw OR ghana:ti,ab,kw OR guinea:ti,ab,kw OR gambia:ti,ab,kw OR grenada:ti,ab,kw OR guatemala:ti,ab,kw OR guyana:ti,ab,kw OR honduras:ti,ab,kw OR haiti:ti,ab,kw OR indonesia:ti,ab,kw OR india:ti,ab,kw OR iran:ti,ab,kw OR iraq:ti,ab,kw OR jamaica:ti,ab,kw OR jordan:ti,ab,kw OR kazakhstan:ti,ab,kw OR kenya:ti,ab,kw OR kyrgyz*:ti,ab,kw OR cambodia:ti,ab,kw OR kiribati:ti,ab,kw OR lao:ti,ab,kw OR laos:ti,ab,kw OR lebanon:ti,ab,kw OR liberia:ti,ab,kw OR libya:ti,ab,kw OR lucia:ti,ab,kw OR 'sri lanka':ti,ab,kw OR lesotho:ti,ab,kw OR morocco:ti,ab,kw OR moldova:ti,ab,kw OR madagascar:ti,ab,kw OR maldives:ti,ab,kw OR mexico:ti,ab,kw OR 'marshall islands':ti,ab,kw OR macedonia:ti,ab,kw OR mali:ti,ab,kw OR myanmar:ti,ab,kw OR montenegro:ti,ab,kw OR mongolia:ti,ab,kw OR mozambique:ti,ab,kw OR mauritania:ti,ab,kw OR malawi:ti,ab,kw OR malaysia:ti,ab,kw OR namibia:ti,ab,kw OR niger:ti,ab,kw OR nigeria:ti,ab,kw OR nicaragua:ti,ab,kw OR nepal:ti,ab,kw OR pakistan:ti,ab,kw OR peru:ti,ab,kw OR philippines:ti,ab,kw OR 'papua new guinea':ti,ab,kw OR 'north korea':ti,ab,kw OR dprk:ti,ab,kw OR paraguay:ti,ab,kw OR 'west bank':ti,ab,kw OR gaza:ti,ab,kw OR russia*:ti,ab,kw OR rwanda:ti,ab,kw OR sudan:ti,ab,kw OR senegal:ti,ab,kw OR 'solomon islands':ti,ab,kw OR 'sierra leone':ti,ab,kw OR 'el salvador':ti,ab,kw OR somalia:ti,ab,kw OR serbia:ti,ab,kw OR 'sao tome':ti,ab,kw OR principe:ti,ab,kw OR suriname:ti,ab,kw OR eswatini:ti,ab,kw OR syria*:ti,ab,kw OR chad:ti,ab,kw OR togo:ti,ab,kw OR thailand:ti,ab,kw OR tajikistan:ti,ab,kw OR turkmenistan:ti,ab,kw OR timor:ti,ab,kw OR tonga:ti,ab,kw OR tunisia:ti,ab,kw OR turkey:ti,ab,kw OR tuvalu:ti,ab,kw OR tanzania:ti,ab,kw OR uganda:ti,ab,kw OR ukraine:ti,ab,kw OR uzbekistan:ti,ab,kw OR grenadines:ti,ab,kw OR venezuela:ti,ab,kw OR vietnam:ti,ab,kw OR vanuatu:ti,ab,kw OR samoa:ti,ab,kw OR kosovo:ti,ab,kw OR yemen:ti,ab,kw OR 'south africa':ti,ab,kw OR zambia:ti,ab,kw OR zimbabwe:ti,ab,kw)
Global Health [b]	(((ab:(Afghanistan or Angola or Albania or Argentina or Armenia or "American Samoa" or Azerbaijan or Burundi or Benin or "Burkina Faso" or Bangladesh or Bulgaria or Bosnia or Herzegovina or Belarus or Belize or Bolivia or Brazil or Bhutan or Botswana or "Central African Republic" or China or "Ivory Coast" or Ivoire or Cameroon or Congo or Colombia or Comoros or "Cabo Verde" or "Costa Rica" or Cuba or Djibouti or Dominica or "Dominican Republic" or Algeria or Ecuador or Egypt or Eritrea or Ethiopia or Fiji or Micronesia or Gabon or Georgia or Ghana or Guinea or Gambia or Grenada or Guatemala or Guyana or Honduras or Haiti or Indonesia or India or Iran or Iraq or Jamaica or Jordan or Kazakhstan or Kenya or Kyrgyz* or Cambodia or Kiribati or Lao or Laos or Lebanon or Liberia or Libya or Lucia or "Sri Lanka" or Lesotho or Morocco or Moldova or Madagascar or Maldives or Mexico or "Marshall Islands" or Macedonia or Mali or Myanmar or Montenegro or Mongolia or Mozambique or Mauritania or Malawi or Malaysia or Namibia or Niger or Nigeria or Nicaragua or Nepal or Pakistan or Peru or Philippines or "Papua New Guinea" or "North Korea" or DPRK or Paraguay or "West Bank" or Gaza or Russia* or Rwanda or Sudan or Senegal or "Solomon Islands" or "Sierra Leone" or "El Salvador" or Somalia or Serbia or "Sao Tome" or Principe or Suriname or Eswatini or Syria* or Chad or Togo or Thailand or Tajikistan or Turkmenistan or Timor or Tonga or Tunisia or Turkey or Tuvalu or Tanzania or Uganda or Ukraine or Uzbekistan or Grenadines or Venezuela or Vietnam or Vanuatu or Samoa or Kosovo or Yemen or "South Africa" or Zambia or Zimbabwe)) OR (title:(Afghanistan or Angola or Albania or Argentina or Armenia or "American Samoa" or Azerbaijan or Burundi or Benin or "Burkina Faso" or Bangladesh or Bulgaria or Bosnia or Herzegovina or Belarus or Belize or Bolivia or Brazil or Bhutan or Botswana or "Central African Republic" or China or "Ivory Coast" or Ivoire or Cameroon or Congo or Colombia or Comoros or "Cabo Verde" or "Costa Rica" or Cuba or Djibouti or Dominica or "Dominican Republic" or Algeria or Ecuador or Egypt or Eritrea or Ethiopia or Fiji or Micronesia or Gabon or Georgia or Ghana or Guinea or Gambia or Grenada or Guatemala or Guyana or Honduras or Haiti or Indonesia or India or Iran or Iraq or Jamaica or Jordan or Kazakhstan or Kenya or Kyrgyz* or Cambodia or Kiribati or Lao or Laos or Lebanon or Liberia or Libya or Lucia or "Sri Lanka" or Lesotho or Morocco or Moldova or Madagascar or Maldives or Mexico or "Marshall Islands" or Macedonia or Mali or Myanmar or Montenegro or Mongolia or Mozambique or Mauritania or Malawi or Malaysia or Namibia or Niger or Nigeria or

	Nicaragua or Nepal or Pakistan or Peru or Philippines or "Papua New Guinea" or "North Korea" or DPRK or Paraguay or "West Bank" or Gaza or Russia* or Rwanda or Sudan or Senegal or "Solomon Islands" or "Sierra Leone" or "El Salvador" or Somalia or Serbia or "Sao Tome" or Principe or Suriname or Eswatini or Syria* or Chad or Togo or Thailand or Tajikistan or Turkmenistan or Timor or Tonga or Tunisia or Turkey or Tuvalu or Tanzania or Uganda or Ukraine or Uzbekistan or Grenadines or Venezuela or Vietnam or Vanuatu or Samoa or Kosovo or Yemen or "South Africa" or Zambia or Zimbabwe))) OR (gl:(Afghanistan or Angola or Albania or Argentina or Armenia or "American Samoa" or Azerbaijan or Burundi or Benin or "Burkina Faso" or Bangladesh or Bulgaria or Bosnia or Herzegovina or Belarus or Belize or Bolivia or Brazil or Bhutan or Botswana or "Central African Republic" or China or "Ivory Coast" or Ivoire or Cameroon or Congo or Colombia or Comoros or "Cabo Verde" or "Costa Rica" or Cuba or Djibouti or Dominica or "Dominican Republic" or Algeria or Ecuador or Egypt or Eritrea or Ethiopia or Fiji or Micronesia or Gabon or Georgia or Ghana or Guinea or Gambia or Grenada or Guatemala or Guyana or Honduras or Haiti or Indonesia or India or Iran or Iraq or Jamaica or Jordan or Kazakhstan or Kenya or Kyrgyz* or Cambodia or Kiribati or Lao or Laos or Lebanon or Liberia or Libya or Lucia or "Sri Lanka" or Lesotho or Morocco or Moldova or Madagascar or Maldives or Mexico or "Marshall Islands" or Macedonia or Mali or Myanmar or Montenegro or Mongolia or Mozambique or Mauritania or Malawi or Malaysia or Namibia or Niger or Nigeria or Nicaragua or Nepal or Pakistan or Peru or Philippines or "Papua New Guinea" or "North Korea" or DPRK or Paraguay or "West Bank" or Gaza or Russia* or Rwanda or Sudan or Senegal or "Solomon Islands" or "Sierra Leone" or "El Salvador" or Somalia or Serbia or "Sao Tome" or Principe or Suriname or Eswatini or Syria* or Chad or Togo or Thailand or Tajikistan or Turkmenistan or Timor or Tonga or Tunisia or Turkey or Tuvalu or Tanzania or Uganda or Ukraine or Uzbekistan or Grenadines or Venezuela or Vietnam or Vanuatu or Samoa or Kosovo or Yemen or "South Africa" or Zambia or Zimbabwe))) AND ((title:(:((risk* or socioeconomic or "social determinant*" or "mental illness*" or poverty or alcohol* or unemploy* or **education** or **wealth** or **employment** or **school** or **university**) or ((substance or drug or drugs) and ("use" or abuse or misuse)))) AND ab:(:((risk* or socioeconomic or "social determinant*" or "mental illness*" or poverty or alcohol* or unemploy* or **education** or **wealth** or **employment** or **school** or **university**) or ((substance or drug or drugs) and ("use" or abuse or misuse))))) AND (title:("violent injur*" or homicide* or "intentional injur*" or murder* or "interpersonal violence" or assault* or "firearm injur*" or "gun violence" or "gang violence" or "penetrating trauma") OR ab:("violent injur*" or homicide* or "intentional injur*" or murder* or "interpersonal violence" or assault* or "firearm injur*" or "gun violence" or "gang violence" or "penetrating trauma")))

[a] Bolded search terms were newly added at the time of the updated search conducted in January 2022

[b] Due to limitations on character length for Global Health search strategies, this search was broken down into multiple queries

Supplemental Table S2: Systematic review inclusion and exclusion criteria

**Category**	**Inclusion**	**Exclusion**
Study Design	Original, peer-reviewed research	Reviews, commentaries, or editorials
		Conference abstract
		Full text not available
	Quantitative study	Qualitative studies
	Individual-level data	Population or ecological studies
		Semi-ecological studies
		Geospatial analyses
		Population-level SES measure
	Analytic study	Descriptive study with no comparison group
	Written in English, Spanish, or Portuguese	Written in another other language
	Published since 1980	Published prior to 1980
Population	LMIC Population	High-income country
		LMIC data grouped with HIC data
	Age 12 or older	Focused only on children <12 years old
Outcome	IPVRI as one of the outcomes studied	No injury outcome
		Outcome includes threats or verbal assault
		Outcome includes all injury types, not specific to IPVRI
		Outcome includes suicide or self-inflicted injury
		Outcome was IPVRI perpetration
		Outcome specific to intimate partner violence, domestic violence, gender-based violence, sexual assault, or rape
		Outcome specific to child abuse or elder abuse
Explanatory variables	Education, employment, income, or wealth data presented for outcome and controls	None of the markers of SES were explored
		Other SDH risk factors, outside our scope
		Alcohol or substances use as only risk factor

Supplemental Table S3: Quality assessment results for included studies [a]

**Author, Year**	**Overall Rating** [b]	**1** [c]	**2** [d]	**3** [e]	**4** [f]	**5** [g]	**6** [h]	**7** [i]	**8** [j]	**9** [k]	**10** [l]	**11** [m]	**12** [n]	**13** [o]	**14** [p]	**15** [q]
Abdalla, 2014	Good	Y	Y	N	N	Y	N	Y	Y	Y	Y	Y	Y	Y	Y	Y
Bachani, 2017	Fair	Y	Y	Y	N	Y	N	CD	Y	Y	Y	Y	N	Y	Y	Y
Bass, 2018	Good	Y	Y	Y	N	Y	Y	Y	Y	Y	Y	Y	Y	N	Y	Y
Bass, 2019	Good	Y	Y	Y	N	Y	Y	Y	Y	Y	Y	Y	Y	N	N	Y
Blair, 2022	Good	Y	Y	Y	N	Y	Y	Y	Y	Y	Y	Y	Y	Y	Y	Y
Borges, 1994	Fair	Y	Y	Y	N	Y	Y	Y	N	Y	Y	Y	Y	Y	Y	CD
Borges, 1998	Fair	Y	Y	Y	N	Y	Y	Y	Y	Y	N	Y	Y	Y	Y	CD
Borges, 2004	Good	Y	Y	Y	N	Y	Y	Y	N	Y	N	Y	Y	Y	Y	CD
Cruz, 2014	Poor	Y	Y	Y	N	CD	N	Y	N	Y	Y	N	CD	CD	N	Y
Doolan, 2007	Good	Y	Y	Y	N	Y	N	Y	Y	Y	Y	N	CD	CD	Y	Y
Duque, 2011	Fair	Y	Y	Y	CD	Y	Y	Y	Y	Y	N	N	Y	CD	Y	Y
Falbo, 2001	Good	Y	Y	Y	Y	Y	Y	Y	Y	Y	Y	Y	N	Y	Y	Y
Fang, 2014	Good	Y	Y	Y	Y	Y	N	Y	Y	Y	Y	N	CD	CD	N	Y
Gathecha, 2018	Good	Y	Y	Y	N	N	Y	Y	Y	Y	Y	Y	CD	Y	Y	Y
Kelly, 2019	Good	Y	Y	Y	N	Y	N	Y	Y	Y	Y	N	Y	CD	Y	Y
Kibusi, 2013	Good	Y	Y	Y	Y	CD	Y	Y	CD	Y	Y	Y	Y	Y	Y	Y
Leeper, 2019	Fair	Y	Y	Y	N	Y	N	Y	N	Y	N	Y	CD	Y	Y	Y
MacDonald, 2005	Fair	Y	N	Y	N	CD	N	Y	CD	Y	N	N	CD	CD	Y	Y
Marchese, 2008	Fair	Y	Y	Y	Y	Y	Y	Y	Y	Y	N	Y	CD	Y	Y	Y
Melo, 2019	Good	Y	Y	Y	N	Y	Y	Y	Y	Y	Y	Y	Y	Y	Y	Y
Mian, 2002	Poor	Y	Y	Y	N	Y	Y	Y	N	Y	N	Y	Y	CD	Y	Y
Orellana, 2017	Good	Y	Y	Y	Y	Y	Y	Y	Y	Y	Y	Y	Y	N	Y	Y
Osaghae, 2020	Good	Y	Y	Y	N	Y	N	Y	Y	Y	Y	Y	CD	Y	Y	Y
Otieno, 2015	Good	Y	Y	Y	N	Y	CD	Y	Y	Y	Y	N	CD	CD	Y	Y
Oyefeso, 2011	Poor	Y	Y	CD	N	Y	N	N	CD	Y	N	N	CD	Y	Y	Y
Purcell, 2020	Good	Y	Y	Y	N	Y	CD	Y	Y	Y	Y	Y	CD	Y	Y	Y
Rubanzana, 2015	Good	Y	Y	Y	N	Y	Y	Y	Y	Y	Y	Y	Y	Y	Y	Y
Salamati, 2015	Poor	Y	Y	Y	CD	Y	N	CD	N	Y	N	Y	Y	Y	Y	Y
Tadesse, 2014	Poor	Y	Y	Y	Y	CD	N	N	N	Y	Y	N	Y	CD	N	Y
Tadesse, 2015	Fair	Y	Y	Y	Y	Y	Y	CD	Y	Y	Y	N	Y	N	N	Y
van der Westhuzien, 2017	Good	Y	Y	Y	Y	Y	CD	Y	N	Y	Y	Y	CD	Y	Y	Y
Yang, 2016	Poor	Y	Y	Y	N	Y	N	Y	N	Y	Y	N	N	N	N	Y
Yang, 2020	Good	Y	Y	Y	Y	CD	Y	Y	Y	Y	Y	Y	Y	Y	Y	Y
Yu, 2020	Good	Y	Y	Y	N	Y	CD	Y	CD	Y	Y	Y	CD	Y	Y	Y

[a] A quality assessment was performed using select items from the National Institutes of Health (NIH) quality assessment tool for observational cohort and cross-sectional studies and the Appraisal tool for cross-sectional studies (AXIS). Responses to each item included yes (Y), no (N), or cannot determine (CD)

[b] After completing all 15 items, each reviewer followed guidance provided by the NIH quality assessment instructions to give an overall quality rating for each article

[c] Were the aims/objectives clear?

[d] Was the target population clearly defined? Is it clear who the research was about?

[e] Were all of the subjects selected or recruited from the same or similar populations?

[f] Was a sample size justification, power description, or variance and effect estimates provided?

[g] Were inclusion and exclusion criteria for being in the study pre-specified and applied uniformly to all participants?

[h] Were measures undertaken to address and categorize non-responders (cross-sectional) or cases and controls that were not included?

[i] Was the outcome (dependent variable) clearly defined, valid, reliable, and implemented consistently across all study participants?

[j] Were the exposure measures (independent variables) clearly defined, valid, reliable, and implemented consistently across all study participants?

[k] Were the methods (including statistical methods) sufficiently described to enable them to be repeated?

[l] Were key potential confounding variables measured and adjusted statistically for their impact on the relationship between exposure and outcome?

[m] Were the basic data (numerators and denominators) for all major findings adequately described?

[n] Was the participation rate (or inclusion percentage for case-control) of eligible persons adequate?

[o] Were the results internally consistent? Is missing data declared, do numbers add up in tables?

[p] Were the results presented for all analyses described in the methods?

[q] Was ethical approval or consent of participants attained?
